# Longitudinal Sampling Reveals Persistence of and Genetic Diversity in Extended-Spectrum Cephalosporin-Resistant *Escherichia coli* From Norwegian Broiler Production

**DOI:** 10.3389/fmicb.2021.795127

**Published:** 2021-12-10

**Authors:** Solveig Sølverød Mo, Madelaine Norström, Jannice Schau Slettemeås, Anne Margrete Urdahl, Amar Anandrao Telke, Marianne Sunde

**Affiliations:** ^1^Section for Food Safety and Animal Health Research, Department of Animal Health, Welfare and Food Safety, Norwegian Veterinary Institute, Oslo, Norway; ^2^Section for Epidemiology, Department of Animal Health, Welfare and Food Safety, Norwegian Veterinary Institute, Oslo, Norway

**Keywords:** *Escherichia coli*, longitudinal, ESC, plasmid, broiler

## Abstract

There are knowledge gaps concerning dynamics of extended-spectrum cephalosporin (ESC)-resistant *Escherichia coli* and their plasmids in broiler production and the persistence of strains on broiler farms. Thus, we aimed at characterising ESC-resistant *Escherichia coli* collected from all flocks reared on 10 different farms during a six-months sampling period. All isolates (*n* = 43) were subjected to whole-genome sequencing, and a subset of isolates (*n* = 7) were also sequenced using oxford nanopore technology and subsequent hybrid assembly in order to do in-depth characterisation of the ESC resistance plasmids. The 43 isolates belonged to 11 different sequence types, and three different ESC resistance gene/plasmid combinations were present, namely, IncK2/*bla*_CMY-2_ (*n* = 29), IncI1/*bla*_CMY-2_ (*n* = 6) and IncI1/*bla*_CTX-M-1_ (*n* = 8). ESC-resistant *E. coli* of different STs and with different ESC resistance gene/plasmid combinations could be present on the same farm, while a single ST and ESC resistance gene/plasmid displaying zero or few SNP differences were present on other farms. In-depth characterisation of IncK2/*bla*_CMY-2_ plasmids revealed that at least two distinct variants circulate in the broiler production. These plasmids showed close homology to previously published plasmids from other countries. Our longitudinal study show that ESC-resistant *E. coli* belong to a multitude of different STs and that different ESC resistance genes and plasmids occur. However, there is also indication of persistence of both ESC-resistant *E. coli* strains and IncK2/*bla*_CMY-2_ plasmids on farms. Further studies are warranted to determine the dynamics of strains, plasmids and ESC resistance genes within single broiler flocks.

## Introduction

In Norway, the situation regarding occurrence of antimicrobial resistance (AMR) in bacteria of animal origin is favourable (EFSA and ECDC, 2020). Moreover, selection pressure from antimicrobial use in Norwegian broiler production is almost non-existing (Animalia, 2015, 2016, 2017, 2018; Refsum, 2015). Despite this, the broiler production has been associated with occurrence of *Escherichia coli* displaying resistance to extended-spectrum cephalosporins (ESC; Sunde et al., 2009; Mo et al., 2014, 2019). ESC-resistant *E. coli* has been introduced with imported breeding material, and subsequently been disseminated through the breeding pyramid (Mo et al., 2014; Myrenås et al., 2018). The odds of a broiler flock being positive have been shown to be significantly higher if the previous flock in the same house was positive (Mo et al., 2016a, 2019). Thus, local re-circulation on farm was suggested as a reason for persistence of ESC-resistant *E. coli* in Norwegian broiler production. However, we have limited knowledge on the genetic relatedness between ESC-resistant *E. coli* isolated from consecutive flocks at the same farm, except the gene encoding ESC resistance (Mo et al., 2016a, 2019).

Thus, in this study, we aimed to do in-depth characterisation of ESC-resistant *E. coli* and their resistance plasmids originating from broiler flocks in houses with multiple positive flocks during a six-months sampling period. This will help us understand the dynamic of ESC-resistant *E. coli* in broiler houses and on farms. Selected isolates were subjected to long-read sequencing and plasmid characterisation in order to investigate the similarity between ESC resistance plasmids present in different *E. coli* multilocus sequence types (STs) from flocks reared in the same broiler house. Detailed knowledge of the plasmids can reveal whether they persist and are able to disseminate between different host strains circulating in the broiler production environment.

## Materials and Methods

### Bacterial Isolates

**Figure 1:**
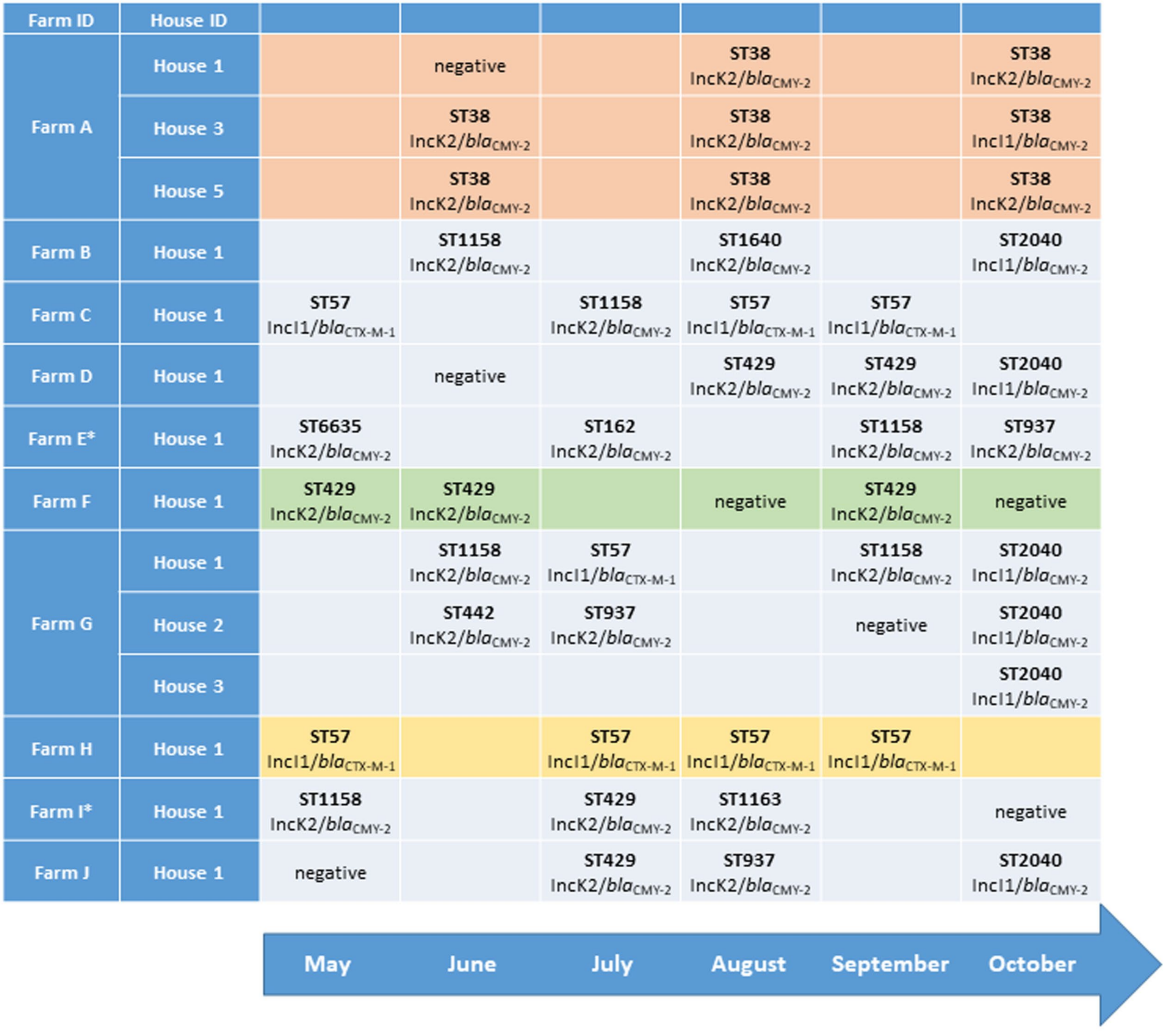
Multilocus sequence type (MLST), plasmid replicon and extended-spectrum cephalosporin (ESC) resistance gene associated with ESC-resistant *Escherichia coli* isolated from different broiler flocks on 10 broiler farms sampled during May–October 2016.

During May–October 2016, boot-swab and dust samples were collected from all broiler flocks reared in Norway (*n* = 2,110). The samples were analysed for the presence of ESC-resistant *Enterobacteriaceae* by plating on MacConkey agar (BD Difco, Beckton, Dickinson and company, Le Pont de Claire, France) supplemented with 1 mg/l cefotaxime (Duchefa, Haarlem, Netherlands) after pre-enrichment in buffered peptone water (BPW-ISO; Mo et al., 2019). This sampling strategy allowed longitudinal sampling on all Norwegian broiler farms (*n* = 701) for a period of 6 months. In the current study, we have included farms with three or more positive flocks (i.e., *E. coli* with plasmid-mediated ESC resistance detected in the flock) in one house during the sampling period. If an included producer had more than one house on the farm, any positive flocks from other houses on the farm were also included. If an included flock was sampled at multiple time points, the first positive sample was included. From each positive flock, one ESC-resistant *E. coli* was included for further analysis. In total, ESC-resistant *E. coli* from 43 unique broiler flocks were included, originating from 14 houses on 10 different broiler farms, named A to J in the following (Figure 1). The genetic background for ESC resistance and minimum inhibitory concentrations for a panel of antimicrobials were determined in a previous study (Mo et al., 2019; Figure 1 and Supplementary Table S1).

### DNA Isolation and Whole-Genome Sequencing

#### Short-Read Sequencing

DNA for short-read sequencing of 35 isolates was extracted using the QIAmp DNA mini kit (Qiagen) according to the manufacturers’ protocol. The remaining eight isolates were sequenced in a previous study (Mo et al., 2020a) and the raw reads were available for inclusion in this study (accession number PRJEB45077). The purity of the DNA was determined on a Nanodrop 2000 (Thermo Fischer Scientific) and the DNA concentration was measured on a Qubit^™^ fluorometer (Thermo Fischer Scientific) using the Qubit dsDNA BroadRange assay kit (Thermo Fischer Scientific). Isolates were prepared with the Nextera Flex library preparation kit (Illumina) and sequenced on an Illumina HiSeq X platform, resulting in 150 bp paired-end reads.

#### Long-Read Sequencing

Isolates from Farms E (*n* = 4) and I (*n* = 3) were subjected to long-read sequencing in order to investigate the similarity between ESC resistance plasmids in *E. coli* of different STs originating from the same farm (Figure 1). DNA was extracted using the Gentra Puregene Yeast/Bact. kit (Qiagen). DNA purity and concentration were determined as described for short-read sequencing. Isolates were prepared with the SQK-RBK004 rapid barcoding kit (Oxford Nanopore Sequencing [Oxford Nanopore Technology], Oxford, United Kingdom) and sequenced using a FLO-MIN106 flow cell on a MinION device (Oxford Nanopore Sequencing).

### *In silico* Analysis

#### Analysis of Short-Read Sequences

The Bifrost pipeline (Lagesen, 2020) was used for initial quality control and assembly of raw reads as previously described (Mo et al., 2020b).

All 43 isolates were further characterised using the ELLIPSIS pipeline (Kaspersen, 2021) with draught assemblies as input. The multilocus sequence type (MLST) was determined for each isolate (Larsen et al., 2012) using the Achtman scheme (Wirth et al., 2006). MOB-suite v 3.0.1 (Robertson and Nash, 2018) was used to predict plasmids and chromosomes, and determine the replicon type for each predicted plasmid. The FASTA files outputted by MOB-suite were annotated using Prokka v 1.14.5 (Seemann, 2014) and used as input to ResFinder v 3.2 (Zankari et al., 2012) and PlasmidFinder v 2.1 (Carattoli et al., 2014) to identify AMR genes and plasmid replicons, respectively. When applicable, plasmid MLST (pMLST) was also determined using pMLST finder v.0.1.0 (Carattoli et al., 2014).

Sequence types represented by at least three isolates were included in single-nucleotide polymorphism (SNP) analysis using Snippy^1^ in order to determine the genetic relatedness between isolates. The fastANI tool (Jain et al., 2018) was used to compare isolates of the same ST to identify a suitable reference to include in the SNP analysis. An isolate with a high similarity to the other isolates belonging to the same ST and good assembly statistics was used as reference.

#### Analysis of Long-Read Sequences

Raw sequence data from the ONT sequencing were prepared as previously described (Kaspersen et al., 2021). Briefly, base calling was done using Guppy v.3.4.5 (Ueno et al., 2003), demultiplexing using qcat v.1.1.0 (ONT^2^) and sequence quality of demultiplexed datasets examined using NanoPlot v 1.30.0 (De Coster et al., 2018).

Both short and long reads were used as input in the ELLIPSIS pipeline (Kaspersen, 2021) for the seven isolates subjected to ONT sequencing. Hybrid assemblies were created using Unicycler v.0.4.8 with bold settings (Wick et al., 2017) after removal of reads <1,000 bp using Filtlong v.0.2.0.^3^ QUAST v.5.0.2 (Gurevich et al., 2013) was used for quality control of assemblies. Thereafter, MOB-suite, Prokka, ResFinder and PlasmidFinder were run as described in the previous section.

To determine if identical or highly similar plasmids were present in *E. coli* of different STs obtained from the same farm, *bla*_CMY-2_ carrying plasmids from the seven ONT sequenced isolates were further compared on farm level. Information regarding number of contigs, total plasmid size (bp), circularity and presence of AMR genes was evaluated (Supplementary Table S2). On Farm E, three plasmids ranging in size from 85,173 to 85,922 bp were compared, while two plasmids of 117,010 bp and 118,095 bp were compared on Farm I. One plasmid from each farm was excluded from the comparisons, as the initial plasmid characterisation showed that the lengths differed from the remaining plasmids on the farms. The plasmid sequences were annotated with Prokka (Seemann, 2014) using the previously published plasmid pDV45 (accession number KR905384) as reference. The annotations were manually curated in CLC Main Workbench 8.0 (CLC bio, Qiagen, Hilden, Germany). Furthermore, we used Snippy^4^ to identify number and location of SNPs in the core genome of the plasmids. For each farm, the gbk file from the longest plasmid, namely, p16449 and p18539, was used as a reference for the Snippy analysis. The plasmid comparisons were visualised using the BRIG (BLAST Ring Image Generator) programme (Alikhan et al., 2011).

## Results

### *E. coli* ST Variability and Genetic Relatedness Between Isolates

In total, 11 different STs were identified among the 43 included isolates (Figure 1). In eight houses (57.1%), all flocks reared during the sampling period were positive for ESC-resistant *E. coli* (Figure 1; Table 1). In five of the 14 houses included in the study (35.7%), all ESC-resistant *E. coli* detected belonged to a single ST throughout the sampling period (Figure 1). Three of these houses were on Farm A, with *E. coli* ST38 present in all flocks (*n* = 8). Eight out of nine (88.9%) flocks reared on Farm A were positive. In the remaining nine houses included (64.3%), we detected ESC-resistant *E. coli* belonging to two-four different STs.

**Table 1:** Overview of characteristics associated with extended-spectrum cephalosporin-resistant *Escherichia coli* originating from 42 broiler flocks reared on 10 broiler farms in Norway during May–October 2016.

Isolate ID	Farm ID	House number	Sampled date	ESC resistance gene	MLST	Plasmid replicon associated with ESC resistance gene (name)	IncI1 pMLST (clonal complex)	Predicted plasmid size (bp) from illumina assembly	Other AMR genes in isolate	ONT sequencing	Predicted plasmid size (bp) from hybrid assembly
2016-40-17710	Farm A	1	22.08.2016	*bla*_CMY-2_	38	IncK2		84,255			
2016-40-20341		1	10.10.2016	*bla*_CMY-2_	38	IncK2		80,064			
2016-40-16014		3	27.06.2016	*bla*_CMY-2_	38	IncK2		85,198			
2016-40-17686		3	22.08.2016	*bla*_CMY-2_	38	IncK2		87,613			
2016-40-20342		3	10.10.2016	*bla*_CMY-2_	38	IncK2^*^		116912^*^	*bla*_TEM-1B_		
2016-40-16007		5	27.06.2016	*bla*_CMY-2_	38	IncK2		81,789			
2016-40-17719		5	22.08.2016	*bla*_CMY-2_	38	IncK2		82,906			
2016-40-20321		5	10.10.2016	*bla*_CMY-2_	38	IncK2		89,953			
2016-40-15014	Farm B	1	01.06.2016	*bla*_CMY-2_	1,158	IncK2		85,440			
2016-40-17027		1	01.08.2016	*bla*_CMY-2_	1,640	IncK2		91,782			
2016-40-20174		1	04.10.2016	*bla*_CMY-2_	2040	IncI1	12	100,533			
2016-40-14497	Farm C	1	23.05.2016	*bla*_CTX-M-1_	57	IncI1	3 (3)	105,215	*sul2*		
2016-40-16264		1	04.07.2016	*bla*_CMY-2_	1,158	IncK2		92,837			
2016-40-17437		1	15.08.2016	*bla*_CTX-M-1_	57	IncI1	3 (3)	102,799	*sul2*		
2016-40-19583		1	26.09.2016	*bla*_CTX-M-1_	57	IncI1	3 (3)	118,271	*sul2*		
2016-40-17018	Farm D	1	01.08.2016	*bla*_CMY-2_	429	IncK2		108,587	*aac*(3)-*VIa*, *aad*A1, *sul1*, *tet*(A)		
2016-40-18913		1	12.09.2016	*bla*_CMY-2_	429	IncK2		74,155	*aac*(3)-*VIa*, *aad*A1, *sul1*, *tet*(A)		
2016-40-21094		1	24.10.2016	*bla*_CMY-2_	2040	IncI1	12	99,795			
2016-40-14821	Farm E	1	30.05.2016	*bla*_CMY-2_	6,635	IncK2 (p14821)		100,051	*bla*_TEM-1B_, *aph*(3″)-Ib, *aph*(6)-Id, *tet*(B)^‡^	+	88,853
2016-40-16852		1	25.07.2016	*bla*_CMY-2_	162	IncK2 (p16852)		90,172		+	85,173
2016-40-18539		1	05.09.2016	*bla*_CMY-2_	1,158	IncK2 (p18539)		101,148		+	85,922
2016-40-20728		1	17.10.2016	*bla*_CMY-2_	937	IncK2 (p20728)		84,450		+	85,922
2016-40-13912	Farm F	1	09.05.2016	*bla*_CMY-2_	429	IncK2		107,423	*aac*(3)-*VIa*, *aad*A1, *sul1*, *tet*(A)		
2016-40-15715		1	20.06.2016	*bla*_CMY-2_	429	IncK2		108,860	*aac*(3)-*VIa*, *aad*A1, *sul1*, *tet*(A)		
2016-40-18912		1	12.09.2016	*bla*_CMY-2_	429	IncK2		108,587	*aac*(3)-*VIa*, *aad*A1, *sul1*, *tet*(A)		
2016-40-15149	Farm G	1	06.06.2016	*bla*_CMY-2_	1,158	IncK2		85,112			
2016-40-16990		1	29.07.2016	*bla*_CTX-M-1_	57	IncI1	3 (3)	103,090	*sul2*		
2016-40-18654		1	05.09.2016	*bla*_CMY-2_	1,158	IncK2		90,829			
2016-40-20530		1	12.10.2016	*bla*_CMY-2_	2040	IncI1	12	100,533			
2016-40-15403		2	13.06.2016	*bla*_CMY-2_	442	IncK2		85,745	*dfr*A5		
2016-40-16989		2	29.07.2016	*bla*_CMY-2_	937	IncK2		88,819			
2016-40-20691		2	17.10.2016	*bla*_CMY-2_	2040	IncI1	12	100,533			
2016-40-19945		3	03.10.2016	*bla*_CMY-2_	2040	IncI1	12	102,212			
2016-40-14272	Farm H	1	18.05.2016	*bla*_CTX-M-1_	57	IncI1	3 (3)	105,345	*sul2*		
2016-40-16262		1	04.07.2016	*bla*_CTX-M-1_	57	IncI1	42 (3)	96,987	*sul2*		
2016-40-17200		1	08.08.2016	*bla*_CTX-M-1_	57	IncI1	42 (3)	93,950	*sul2*		
2016-40-19738		1	28.09.2016	*bla*_CTX-M-1_	57	IncI1	42 (3)	112,625	*sul2*		
2016-40-14849	Farm I	1	30.05.2016	*bla*_CMY-2_	1,158	IncK2 (p14849)		101,723		+	84,386
2016-40-16449		1	11.07.2016	*bla*_CMY-2_	429	IncK2 (p16449)		110,605	*aac*(3)-*VIa*, *aad*A1, *sul1*, *tet*(A)	+	118,095
2016-40-17695		1	22.08.2016	*bla*_CMY-2_	1,163	IncK2 (p17695)		106,308	*aac*(3)-*VIa*, *aad*A1, *sul1*, *tet*(A), *fos*A7	+	117,010
2016-40-16572	Farm J	1	13.07.2016	*bla*_CMY-2_	429	IncK2		107,681	*aac*(3)-*VIa*, *aad*A1, *sul1*, *tet*(A)		
2016-40-18061		1	29.08.2016	*bla*_CMY-2_	937	IncK2		97,428			
2016-40-20306		1	10.10.2016	*bla*_CMY-2_	2040	IncI1	12	100,533			

*ONT, Oxford Nanopore Technology; MLST, multilocus sequence type; and ESC, extended-spectrum cephalosporin;*

* *bla_CMY-2_ probably located on an IncK2 plasmid, suggested length might be erroneous;*

‡ *AMR genes not located on the IncK2 plasmid*.

All ST38 isolates (*n* = 8) originated from flocks reared in three different houses on Farm A. The SNP analysis showed that the isolates differed by 6–55 SNPs, with 13 or more SNP differences between six of the eight isolates. *E. coli* ST57 (*n* = 8) was detected from three different farms and differed by 0–21 SNPs. Three ST57 isolates from Farm C displayed no SNP differences, while four isolates from Farm H differed by 1–6 SNPs. Two ST429 isolates from Farm D differed by 6 SNPs, while three isolates from Farm F differed by 2–6 SNPs. ST1158 isolates were repeatedly recovered only from House 1 on Farm G, and 23 SNP differences were present between the isolates. In addition, *E. coli* ST1158 were isolated from four other farms, displaying up to 120 SNP differences. Isolates belonging to ST2040 were only detected in the last sampling month, and consecutive isolates from the same farm were therefore not obtained. Three ST2040 isolates were from three different houses on Farm G, and these isolates differed by 4–8 SNPs. Three ST937 isolates from three different farms differed by 23–37 SNPs. An overview of SNP differences present between isolates from the same ST is presented in the supplementary material (Supplementary Tables S3A-F).

### Co-resistance to Other Antimicrobials

In 24 (55.8%) of the included isolates, *bla*_CMY-2_ was the only AMR gene detected. The most common co-resistance observed among the isolates was sulfamethoxazole resistance (*n* = 16, 37.2%) encoded by either *sul1* in *bla*_CMY-2_ carrying isolates (*n* = 8, 18.6%) or *sul2* in *bla*_CTX-M-1_ carrying isolates (*n* = 8, 18.6%). Also, co-resistance towards aminoglycosides (*n* = 9, 20.9%) or tetracycline (*n* = 8, 18.6%) was observed. Aminoglycoside resistance was associated with the presence of *aac* and *aad* (*n* = 8, 18.6%) or *aph* (*strA*/*strB*) genes (*n* = 1, 2.3%), while tetracycline resistance was encoded by *tet*(A) (*n* = 8, 18.6%) or *tet*(B) (*n* = 1, 2.3%; Table 1).

### ESC Resistance Plasmids

The majority of isolates (*n* = 35, 81.4%) carried the *bla*_CMY-2_ gene encoding ESC resistance. *bla*_CMY-2_ was frequently associated with IncK2 plasmids (*n* = 29) but was also found on IncI1 plasmids (*n* = 6). In eight isolates (18.6%), ESC resistance was mediated by *bla*_CTX-M-1_ (Figure 1; Table 1). The *bla*_CTX-M-1_ gene was previously found to be associated with IncI1-Iγ plasmids in all isolates (Mo et al., 2020b). IncK2/*bla*_CMY-2_ plasmids were detected in nine different STs, with a predicted size of 74–110 kb. IncI1/*bla*_CMY-2_ plasmids and IncI1/*bla*_CTX-M-1_ plasmids were only found in unique STs, namely, ST2040 and ST57, respectively. The predicted lengths were approximately 100 kb for IncI1/*bla*_CMY-2_ plasmids and 94–118 kb for IncI1/*bla*_CTX-M-1_ plasmids (Figure 1; Table 1). In a single ST38 isolate (2016-40-20342), MOB-suite predicted *bla*_CMY-2_ to be located on an IncI1 plasmid. The contig encoding *bla*_CMY-2_ was subjected to a BLAST search and showed 100% identity to previously published IncK2 plasmid sequences. Thus, we assumed that it was erroneously classified as an IncI1 plasmid and that *bla*_CMY-2_ was associated to an IncK2 plasmid in this isolate.

In order to investigate possible on farm plasmid persistence and transfer between *E. coli* host strains, we performed detailed characterisation of selected ESC resistance plasmids. All isolates from Farms E and I (*n* = 7) were subjected to long-read sequencing and subsequent analysis. Farms E and I were chosen since IncK plasmids with *bla*_CMY-2_ were present in *E. coli* belonging to four and three different STs, respectively (Figure 1; Table 1). Hybrid assemblies with subsequent plasmid characterisation and comparison revealed that all four isolates on Farm E harboured IncK2 plasmids with *bla*_CMY-2_ as the only AMR gene, ranging from 85,173 to 88,853 bp in size (Table 1). The plasmids were named p14821, p16852, p18539 and p20728. Plasmids p18539 and p20728 from Farm E differed by eight SNPs. One SNP was in the region encoding the *relE* gene, five SNPs in non-coding regions and two SNPs in regions encoding uncharacterized proteins (YfeC and YffA). Between p16852 and p18539, 15 SNPs were present in the core genome (98.1%). Four of the SNPs were in regions encoding uncharacterized proteins (YfaB), five SNPs in non-coding sequences, four SNPs were in the impCAB operon, one SNP in the *relE* gene and one in the *traH* gene. Plasmids p16852 and p20729 differed by four SNPs in the impCAB operon, one SNP in the *traH* gene and six SNPs in regions encoding uncharacterized proteins (YfaB, YfeC and YffA), a total of 11 SNPs (Supplementary Tables S4A,B). In two isolates from Farm I, longer IncK2 plasmids were present (117010–118,095 bp), namely, p16449 and p17695. These plasmids harboured *aac(3)VIa*, *aadA1*, *sul1* and *tet*(A) in addition to *bla*_CMY-2_ (Table 1). No SNP differences were present in the core genome (98.2%) between these two plasmids. The third plasmid from Farm I, named p14849, only harboured *bla*_CMY-2_ and was 84,386 bp long. The initial plasmid characterisation revealed that p1821 (Farm E) and p14849 (Farm I) were of different length compared to the remaining plasmids from the farm, and they were therefore excluded from the SNP analyses and visual comparison.

**Figure 2:**
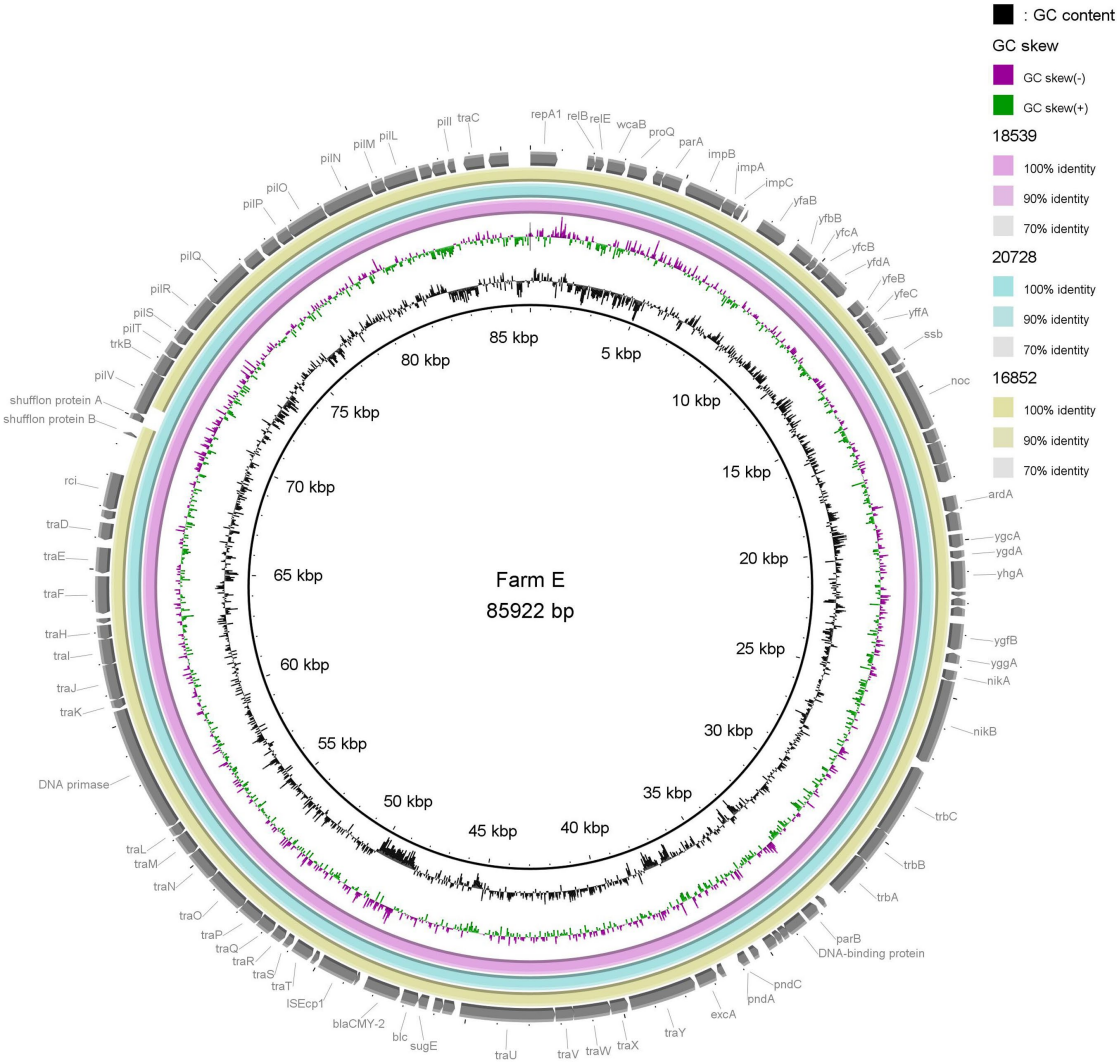
Comparison of three IncK2/*bla*_CMY-2_ plasmids (85–86 kb) originating from extended-spectrum cephalosporin-resistant *Escherichia coli* of three different sequence types isolated from three different flocks on a single farm. Plasmid p18539 was used as reference.

**Figure 3:**
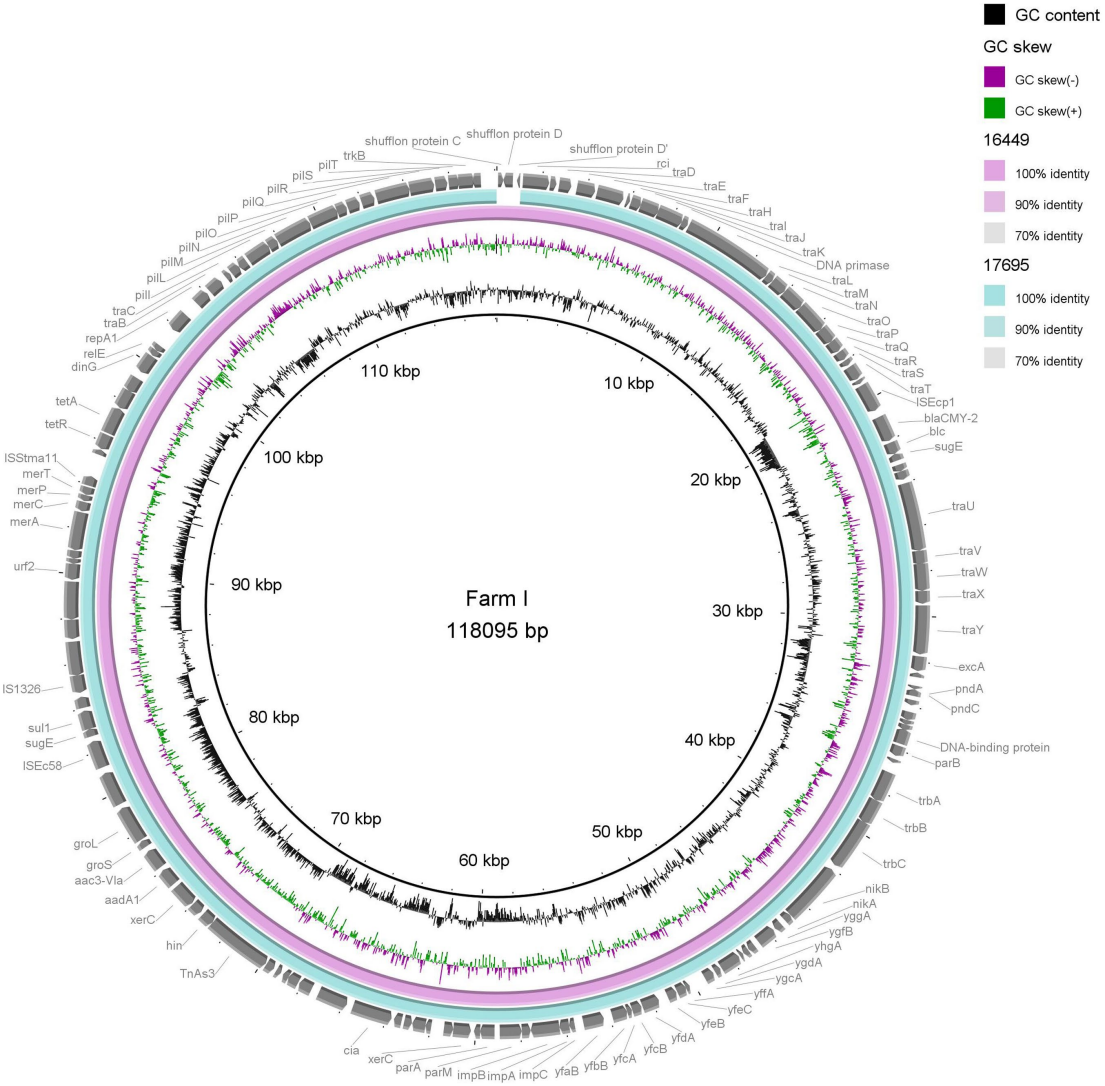
Comparison of two IncK/*bla*_CMY-2_ plasmids (117–118 kb) originating from extended-spectrum cephalosporin-resistant *Escherichia coli* of two different sequence types isolates from two different flocks on the same farm. Plasmid p16449 was used as reference.

Visual comparisons between the three plasmids from Farm E (p16852, p18539 and p20728) and two plasmids from Farm I (p16449 and p17695), respectively, underlined the close genetic relationship between IncK2/*bla*_CMY-2_ plasmids present in different *E. coli* STs on the same farm (Figures 2, 3). The shufflon region differed among the plasmids, while the remaining parts were highly similar.

BLAST revealed close sequence homology between plasmid pESBL3310 (acc. no. MW390545.1) originating from retail chicken meat in Netherlands and the two plasmids p16449 and p17695 (approx. 117–118 kb) from Farm E. Close homology was also found between pDV45 (broiler, Switzerland, acc. no. KR905384.1), p23C16–2 (broiler chicken, Japan, acc. no. LC501559) and the shorter (approx. 85–86 kb) IncK2 plasmids from Farms E and I (data not shown).

Raw reads are available from the European Nucleotide Archive (ENA; accession number PRJEB47914 for Illumina reads and PRJEB48023 for Oxford Nanopore reads).

## Discussion

In this study, we have analysed ESC-resistant *E. coli* from Norwegian broiler farms sampled longitudinally during a six-month period in 2016. Our overall results indicated genetic diversity at strain level and a multitude of circulating ESC-resistant strains in broiler production, but also potential persistence of ESC-resistant *E. coli* on farms exemplified by ST57 on Farms C and H, and ST429 on Farms D and F. Although the occurrence of ESC-resistant *E. coli* is currently very low (0.4%) in Norwegian broiler production (NORM/NORM-VET, 2021), we consider this as valuable knowledge that can be extrapolated to other AMR *E. coli*.

On Farm A, we found ESC-resistant *E. coli* ST38 in all sampled flocks. Isolates from flocks reared in the same house were not necessarily more similar than isolates from flocks reared in different houses. Furthermore, up to 55 SNPs between isolates could indicate that *E. coli* ST38 has been introduced to the farm on several time points, rather than one strain persisting on the farm. For *E. coli* ST2040, ST429 and ST57, highly similar isolates were present in different flocks on the same farms, but also on different farms. For example, ST57 isolates originating from Farm C differed by zero SNPs and isolates from Farm H differed by one-six SNPs. This indicates potential clonal dissemination of these STs, for example from supplying parent flock(s) and that they may have the capability of persisting on broiler farms and/or in broiler houses between production rounds. ST57 isolates were overall more diverse than ST2040 and ST429 isolates. This could be due to previous introduction to the different farms followed by persistence and local evolution of the strain, or circulation of several ST57 strains in the broiler production. The same was observed for three ST937 isolates. However, we do not have any historical isolates available for comparison, and the reason for the observed diversity therefore remains undetermined. *E. coli* ST1158 was present on five different farms. At least two different strains were circulating, as up to 120 SNPs were observed. We have previously described the presence of both *E. coli* ST38 and ST1158 with *bla*_CMY-2_ in Norwegian broiler production (Mo et al., 2016b; Buberg et al., 2021). The first ST38 isolate described was from 2011, while ST1158 emerged in 2014 (Mo et al., 2016b). On the contrary, *E. coli* ST2040 and ST429 were first described from Norwegian broiler production in 2016 and were collected as part of the Norwegian monitoring programme on AMR in the veterinary sector, NORM-VET (Buberg et al., 2021). MLST has not been performed routinely on ESC-resistant *E. coli* collected in NORM-VET, but we have characterised all ESC-resistant isolates from retail chicken meat (2012–2016) from this programme (Buberg et al., 2021). Thus, it is reasonable to assume that ST38 and ST1158 circulated in the Norwegian broiler production for several years, while ST2040 and ST429 were introduced shortly before or during 2016. This could also be part of the explanation for the higher number of SNPs present among ST38 and ST1158 isolates compared to ST2040 and ST429 isolates.

ESC-resistant *E. coli* belonging to different STs were present in most included houses, indicating limited clonal persistence in the production. In addition, we detected different ESC resistance genes and plasmids in some of the houses. However, it was not possible to determine whether different genotypes were present simultaneously in a flock, as we only characterised one isolate per sample. Persistence of ESC-resistant strains could therefore be more common than suggested here.

In-depth characterisation revealed that three isolates of different STs from Farm E harboured highly similar IncK2/*bla*_CMY-2_ plasmids of approximately 85–86 kb. Furthermore, two isolates of different STs from Farm I carried IncK2/*bla*_CMY-2_ plasmids of approximately 117–118 kb with no SNP differences. This suggests possible horizontal transfer of and/or on farm persistence of plasmids. We have previously shown that most IncK/*bla*_CMY-2_ plasmids from Norwegian broiler production are self-transferable (Mo et al., 2016b). Thus, it is possible that IncK2/*bla*_CMY-2_ plasmids have transferred between different *E. coli* STs, resulting in dissemination and persistence of ESC resistance in the production pyramid, as suggested by others (Baron et al., 2018; van Hoek et al., 2018). We have previously demonstrated transfer of IncK/*bla*_CMY-2_ plasmids into *Serratia* spp. and back to *E. coli*. This suggests a potential plasmid reservoir in environmental bacteria, possibly affecting their ability to persist in broiler production (Mo et al., 2017).

On Farm I, two of the characterised isolates harboured a long (approx. 117–118 kb) IncK2/*bla*_CMY-2_ plasmid, while the third isolate harboured a shorter version (~84 kb). Thus, we can assume that distinct variants of IncK2/*bla*_CMY-2_ plasmids are circulating in the Norwegian broiler production. Based on our strategy of analysing a single isolate per flock, it was not possible to evaluate whether the two IncK2/*bla*_CMY-2_ plasmid variants were simultaneously present on Farm I. According to analyses done in MOB-suite using short-read data, the IncK2/*bla*_CMY-2_ plasmids included in this study varied from 74 to 110 kb, indicating that more than two variants are circulating. However, long-read sequencing and subsequent hybrid assemblies are necessary to get a more reliable prediction of plasmid length. The IncK2/*bla*_CMY-2_ plasmids characterised in the present study showed high sequence homology to previously published plasmids originating from broiler production in Switzerland, Netherlands and Japan. This indicated the presence of endemic plasmids circulating internationally in broiler production, as previously suggested (Mo et al., 2017; Seiffert et al., 2017).

Interestingly, all six *E. coli* ST2040 were from flocks reared in October 2016 on three farms. These harboured IncI1/*bla*_CMY-2_ plasmids, whereas only IncK2/*bla*_CMY-2_ and/or IncI1/*bla*_CTX-M-1_ plasmids were detected on these farms previously. IncI1/*bla*_CMY-2_ plasmids from ST2040 are non-transferable *in vitro*. Also, ST2040 was detected for the first time in ESC-resistant *E. coli* from Norwegian retail chicken meat in 2016 (Buberg et al., 2021). This, together with the limited number of SNPs (3–12), could indicate a common source of the ST2040 isolates, and potential vertical dissemination in the production pyramid. However, this could not be confirmed as information regarding hatchery of origin and supplying parent flocks was not available. It was not possible to determine if ST2040 could persist in the production, as the final samples were collected in October 2016.

It is possible that the observed diversity among ESC-resistant *E. coli* is in fact a reflection of the most abundant *E. coli* STs present in the broiler gut, but further studies are warranted to confirm this. It has been suggested that several isolates should be investigated in order to reflect the diversity of ESC-resistant *E. coli* in a sample (van Hoek et al., 2018). If susceptible *E. coli* were characterised in parallel, it would substantially increase the knowledge regarding within-flock diversity of susceptible and resistant *E. coli*, but also regarding transmission dynamics of ESC resistance in broiler flocks.

To our best knowledge, this is the first longitudinal study on ESC resistance in *E. coli* from broilers using both short- and long-read sequencing technology, enabling in-depth characterisation of isolates and resistance plasmids. Our results indicate diversity on strain-, plasmid- and ESC resistance gene level. In conclusion, this study has increased our knowledge regarding the dynamics and diversity of ESC-resistant *E. coli* in Norwegian broiler production. Seemingly, local persistence and re-circulation of ESC-resistant *E. coli* strains happen. However, different *E. coli* STs and even different plasmids carrying ESC resistance were observed in some houses/farms. We further describe the presence of highly similar plasmids in different *E. coli* STs, which could be due to persistence of ESC resistance plasmids. As we only characterised one isolate per positive flock, we have no knowledge regarding the composition of the ESC-resistant *E. coli* population. Thus, it is possible that several different *E. coli* STs carrying ESC resistance plasmids are present simultaneously. In addition, it is possible that some *E. coli* STs have the ability to persist between production cycles. Further exploration of the diversity of *E. coli* STs (susceptible and resistant) should be done in order to increase our understanding of the dynamics of ESC-resistant *E. coli*.

## Data Availability Statement

The datasets presented in this study can be found in online repositories. The names of the repository/repositories and accession number(s) can be found in the article/Supplementary Material.

## Author Contributions

SM, MN, and MS contributed to the conception and design of the present study, while AU was responsible for the conception and design of the study the isolates originated from. SM, JS, and AT performed the *in silico* analyses. SM wrote the first draft of the manuscript. All authors contributed to manuscript revision, read, and approved the submitted version.

## Funding

This work was supported by funding from the Norwegian Veterinary Institute and the European Union’s Horizon 2020 Research and Innovation programme under grant agreement no 773830: One Health European Joint Programme, OH-EJP-H2020-JRP-AMR-2-ARDIG. Parts of the data were presented during the One Health EJP Annual Scientific Meeting in Dublin, May 2019.

## Conflict of Interest

The authors declare that the research was conducted in the absence of any commercial or financial relationships that could be construed as a potential conflict of interest.

## Publisher’s Note

All claims expressed in this article are solely those of the authors and do not necessarily represent those of their affiliated organizations, or those of the publisher, the editors and the reviewers. Any product that may be evaluated in this article, or claim that may be made by its manufacturer, is not guaranteed or endorsed by the publisher.

## Footnotes

^1^ https://github.com/tseemann/snippy

^2^ https://github.com/nanoporetech/qcat

^3^ https://github.com/rrwick/Filtlong

^4^ https://github.com/tseemann/snippy
