# Supplementary material for: Longitudinal Sampling Reveals Persistence of and Genetic Diversity in Extended-Spectrum Cephalosporin-Resistant Escherichia coli From Norwegian Broiler Production
Source: Front Microbiol. 2021 Dec 10;12:795127. doi: 10.3389/fmicb.2021.795127 (PMC8702822; doi:10.3389/fmicb.2021.795127)
Supplement: Supplementary file 1 [file Data_Sheet_1.docx]

Supplementary Material

# Supplementary Figures and Tables

## Supplementary Tables

**Supplementary Table 1.** Minimum inhibitory concentrations (MICs) and antimicrobial resistance in extended-spectrum cephalosporin-resistant *Escherichia coli* isolated from broiler flocks (n=43) on ten Norwegian broiler farms during May-October 2016.

| Substance | Resistance (n) | Distribution (n) of MIC values (mg/L)* | | | | | | | | | | | | | | | |
| --- | --- | --- | --- | --- | --- | --- | --- | --- | --- | --- | --- | --- | --- | --- | --- | --- | --- |
|  |  | 0.015 | 0.03 | 0.06 | 0.12 | 0.25 | 0.5 | 1 | 2 | 4 | 8 | 16 | 32 | 64 | 128 | 256 | ≥512 |
| Tetracycline | 8 |  |  |  |  |  |  |  | 34 | 1 |  |  |  | 7 | 1 |  |  |
| Tigecycline | 0 |  |  |  |  | 43 |  |  |  |  |  |  |  |  |  |  |  |
| Chloramphenicol | 0 |  |  |  |  |  |  |  |  |  | 42 | 1 |  |  |  |  |  |
| Ampicillin | 43 |  |  |  |  |  |  |  |  |  |  |  |  |  | 43 |  |  |
| Cefotaxime | 43 |  |  |  |  |  |  |  |  | 1 | 42 |  |  |  |  |  |  |
| Ceftazidime | 43 |  |  |  |  |  |  | 1 | 6 | 1 | 2 | 15 |  |  |  |  |  |
| Meropenem | 0 |  | 43 |  |  |  |  |  |  |  |  |  |  |  |  |  |  |
| Sulfamethoxazole | 15 |  |  |  |  |  |  |  |  |  | 28 |  |  |  |  |  | 15 |
| Trimethoprim | 1 |  |  |  |  | 42 |  |  |  |  |  |  |  | 1 |  |  |  |
| Azithromycin | 0 |  |  |  |  |  |  |  | 11 | 28 | 4 |  |  |  |  |  |  |
| Gentamicin | 8 |  |  |  |  |  | 3 | 4 | 1 |  | 1 | 5 | 2 |  |  |  |  |
| Ciprofloxacin | 3 | 36 | 3 | 1 |  | 2 | 1 |  |  |  |  |  |  |  |  |  |  |
| Nalidixic acid | 3 |  |  |  |  |  |  |  |  | 40 |  |  |  |  | 1\| | 2 |  |
| Colistin | 0 |  |  |  |  |  |  | 43 |  |  |  |  |  |  |  |  |  |

*Bold vertical lines denote epidemiological cut-off values for resistance. White fields denote range of dilutions tested for each antimicrobial agent. MIC values higher than the highest concentration tested are given as the lowest MIC value above the range. MIC values equal to or lower than the lowest concentration tested are given as the lowest concentration tested.

**Supplementary Table 2.** Overview of characteristics associated with IncK2/*bla*_CMY-2_ plasmids from *Escherichia coli* isolated from seven different broiler flocks on two farms in Norway during May-October 2016.

| Farm | isolate ID | element | contigs | total_size | closed | CDS | gene | rep_type(s) | plasmidfinder_replicon | resfinder_gene(s) | comment |
| --- | --- | --- | --- | --- | --- | --- | --- | --- | --- | --- | --- |
| E | 2016-40-14821 | p14821 | 1 | 88853 | TRUE | 108 | 108 | IncK2/Z | IncB/O/K/Z | *bla*_CMY-2_ | not included in SNP analysis |
| E | 2016-40-16852 | p16852 | 1 | 85173 | FALSE | 102 | 102 | IncK2/Z | IncB/O/K/Z | *bla*_CMY-2_ |  |
| E | 2016-40-18539 | p18539 | 1 | 85922 | TRUE | 104 | 104 | IncK2/Z | IncB/O/K/Z | *bla*_CMY-2_ | reference in SNP analysis |
| E | 2016-40-20728 | p20728 | 1 | 85922 | TRUE | 104 | 104 | IncK2/Z | IncB/O/K/Z | *bla*_CMY-2_ |  |
| I | 2016-40-14849 | p14849 | 3 | 84386 | FALSE | 100 | 100 | IncK2/Z | IncB/O/K/Z | *bla*_CMY-2_ | not included in SNP analysis |
| I | 2016-40-16449 | p16449 | 1 | 118095 | FALSE | 139 | 139 | IncK2/Z | IncB/O/K/Z | *aac*(3)-VIa,*aad*A1, *bla*_CMY-2_,*sul1*,*tet*(A) | reference in SNP analysis |
| I | 2016-40-17695 | p17695 | 1 | 117010 | FALSE | 136 | 136 | IncK2/Z | IncB/O/K/Z | *aac*(3)-VIa,*aad*A1, *bla*_CMY-2_,*sul1*,*tet*(A) |  |

**Supplementary Tables 3a-f.** Number of single nucleotide polymorphisms (SNPs) present in the core genome between pairs of *Escherichia coli* isolates belonging to the same multilocus sequence type. The isolates used as references are highlighted with bold font.

**a) ST38**

| snp-dists 0.6.3 | Farm A, House 5, Flock 1 | **Farm A, House 3, Flock 2** | Farm A, House 1, Flock 1 | Farm A, House 5, Flock 2 | Farm A, House 1, Flock 2 | Farm A, House 3, Flock 3 | Farm A, House 5, Flock 3 | Farm A, House 3, Flock 1 | Reference |
| --- | --- | --- | --- | --- | --- | --- | --- | --- | --- |
| Farm A, House 5, Flock 1 | 0 | 35 | 35 | 41 | 43 | 28 | 41 | 33 | 36 |
| **Farm A, House 3, Flock 2** | 35 | 0 | 43 | 49 | 17 | 40 | 15 | 6 | 0 |
| Farm A, House 1, Flock 1 | 35 | 43 | 0 | 44 | 49 | 32 | 49 | 41 | 44 |
| Farm A, House 5, Flock 2 | 41 | 49 | 44 | 0 | 55 | 38 | 55 | 47 | 49 |
| Farm A, House 1, Flock 2 | 43 | 17 | 49 | 55 | 0 | 42 | 24 | 15 | 17 |
| Farm A, House 3, Flock 3 | 28 | 40 | 32 | 38 | 42 | 0 | 42 | 34 | 44 |
| Farm A, House 5, Flock 3 | 41 | 15 | 49 | 55 | 24 | 42 | 0 | 13 | 15 |
| Farm A, House 3, Flock 1 | 33 | 6 | 41 | 47 | 15 | 34 | 13 | 0 | 6 |
| Reference | 36 | 0 | 44 | 49 | 17 | 44 | 15 | 6 | 0 |

*core genome 97.5%, reference 2016-40-17686 (bold font)

**b) ST429**

| snp-dists 0.6.3 | Farm F, Flock 1 | Farm D, Flock 1 | Farm I, Flock 2 | **Farm F, Flock 3** | Farm D, Flock 2 | Farm J, Flock 1 | Farm F, Flock 2 | Reference |
| --- | --- | --- | --- | --- | --- | --- | --- | --- |
| Farm F, Flock 1 | 0 | 3 | 2 | 3 | 3 | 2 | 2 | 4 |
| Farm D, Flock 1 | 3 | 0 | 5 | 6 | 6 | 5 | 5 | 7 |
| Farm I, Flock 2 | 2 | 5 | 0 | 5 | 5 | 4 | 5 | 6 |
| **Farm F, Flock 3** | 3 | 6 | 5 | 0 | 6 | 5 | 6 | 0 |
| Farm D, Flock 2 | 3 | 6 | 5 | 6 | 0 | 5 | 6 | 7 |
| Farm J, Flock 1 | 2 | 5 | 4 | 5 | 5 | 0 | 5 | 6 |
| Farm F, Flock 2 | 2 | 5 | 5 | 6 | 6 | 5 | 0 | 7 |
| Reference | 4 | 7 | 6 | 0 | 7 | 6 | 7 | 0 |

*core genome 99.9%, reference 2016-40-18912 (bold font)

**c) ST1158**

| snp-dists 0.6.3 | Farm C, Flock 2 | **Farm G, House 1, Flock 1** | Farm G, House 1, Flock 3 | Farm E, Flock 3 | Farm B, Flock 1 | Farm I, Flock 1 | Reference |
| --- | --- | --- | --- | --- | --- | --- | --- |
| Farm C, Flock 2 | 0 | 21 | 15 | 10 | 112 | 13 | 22 |
| **Farm G, House 1, Flock 1** | 21 | 0 | 23 | 18 | 120 | 23 | 0 |
| Farm G, House 1, Flock 3 | 15 | 23 | 0 | 13 | 116 | 17 | 23 |
| Farm E, Flock 3 | 10 | 18 | 13 | 0 | 109 | 12 | 20 |
| Farm B, Flock 1 | 112 | 120 | 116 | 109 | 0 | 115 | 120 |
| Farm I, Flock 1 | 13 | 23 | 17 | 12 | 115 | 0 | 23 |
| Reference | 22 | 0 | 23 | 20 | 120 | 23 | 0 |

*core genome 98.4%, reference 2016-40-15149 (bold font)

**d) ST57**

| snp-dists 0.6.3 | Farm H, Flock 3 | Farm C, Flock 3 | Farm C, Flock 4 | Farm H, Flock 4 | Farm H, Flock 1 | Farm C, Flock 1 | **Farm H, Flock 2** | Farm G, House 1, Flock 2 | Reference |
| --- | --- | --- | --- | --- | --- | --- | --- | --- | --- |
| Farm H, Flock 3 | 0 | 20 | 20 | 2 | 5 | 20 | 1 | 13 | 5 |
| Farm C, Flock 3 | 20 | 0 | 0 | 21 | 17 | 0 | 19 | 13 | 27 |
| Farm C, Flock 4 | 20 | 0 | 0 | 20 | 17 | 0 | 19 | 13 | 28 |
| Farm H, Flock 4 | 2 | 21 | 20 | 0 | 6 | 20 | 1 | 14 | 6 |
| Farm H, Flock 1 | 5 | 17 | 17 | 6 | 0 | 17 | 4 | 10 | 12 |
| Farm C, Flock 1 | 20 | 0 | 0 | 20 | 17 | 0 | 19 | 13 | 28 |
| **Farm H, Flock 2** | 1 | 19 | 19 | 1 | 4 | 19 | 0 | 12 | 3 |
| Farm G, House 1, Flock 2 | 13 | 13 | 13 | 14 | 10 | 13 | 12 | 0 | 21 |
| Reference | 5 | 27 | 28 | 6 | 12 | 28 | 3 | 21 | 0 |

*core genome 96.6%, reference 2016-40-16262 (bold font)

**e) ST937**

| snp-dists 0.6.3 | Farm E, Flock 4 | Farm G, House 2, Flock 2 | **Farm J, Flock 2** | Reference |
| --- | --- | --- | --- | --- |
| Farm E, Flock 4 | 0 | 23 | 23 | 27 |
| Farm G, House 2, Flock 2 | 23 | 0 | 37 | 52 |
| **Farm J, Flock 2** | 23 | 37 | 0 | 4 |
| Reference | 27 | 52 | 4 | 0 |

*core genome 98.2%, reference 2016-40-18061 (bold font)

**f) ST2040**

| snp-dists 0.6.3 | Farm B, Flock 3 | Farm G, House 3, Flock 1 | **Farm J, Flock 3** | Farm G, House 1, Flock 4 | Farm G, House 2, Flock 3 | Farm D, Flock 3 | Reference |
| --- | --- | --- | --- | --- | --- | --- | --- |
| Farm B, Flock 3 | 0 | 4 | 5 | 6 | 11 | 8 | 5 |
| Farm G, House 3, Flock 1 | 4 | 0 | 3 | 4 | 8 | 6 | 3 |
| **Farm J, Flock 3** | 5 | 3 | 0 | 5 | 9 | 7 | 0 |
| Farm G, House 1, Flock 4 | 6 | 4 | 5 | 0 | 7 | 8 | 5 |
| Farm G, House 2, Flock 3 | 11 | 8 | 9 | 7 | 0 | 12 | 9 |
| Farm D, Flock 3 | 8 | 6 | 7 | 8 | 12 | 0 | 10 |
| Reference | 5 | 3 | 0 | 5 | 9 | 10 | 0 |

*core genome 99.6%, reference 2016-40-20306 (bold font)

**Supplementary Tables 4a-b.** Number of single nucleotide polymorphisms (SNPs) present in the core genome between pairs of IncK2/*bla*_CMY-2_ plasmids. The plasmids used as references are highlighted with bold font.

a) Short IncK2/*bla*_CMY-2_ plasmids from Farm E

| snp-dists 0.6.3 | p16852 | **p18539** | p20728 | Reference |
| --- | --- | --- | --- | --- |
| p16852 | 0 | 15 | 11 | 15 |
| **p18539** | 15 | 0 | 8 | 0 |
| p20728 | 11 | 8 | 0 | 8 |
| Reference | 15 | 0 | 8 | 0 |

*core genome 98.1%, p18539 used as reference (bold font)

b) Long IncK2/*bla*_CMY-2_ plasmids from Farm I

| snp-dists 0.6.3 | **p16449** | p17695 | Reference |
| --- | --- | --- | --- |
| **p16449** | 0 | 0 | 0 |
| p17695 | 0 | 0 | 0 |
| Reference | 0 | 0 | 0 |

*core genome 98.2%, p16449 used as reference (bold font)
